# EMAT: Enhanced Multi-Aspect Attention Transformer for Financial Time Series Forecasting

**DOI:** 10.3390/e27101029

**Published:** 2025-10-01

**Authors:** Yingjun Chen, Wenfeng Shen, Han Liu, Xiaolin Cao

**Affiliations:** School of Computer and Information Engineering, Shanghai Polytechnic University, Shanghai 201209, China

**Keywords:** financial time series prediction, Multi-Aspect Attention Mechanism, transformer model, volatility-aware modeling, stock market forecasting

## Abstract

Financial time series prediction remains a challenging task due to the inherent non-stationarity, noise, and complex temporal dependencies present in market data. Traditional forecasting methods often fail to capture the multifaceted nature of financial markets, where temporal proximity, trend dynamics, and volatility patterns simultaneously influence price movements. To address these limitations, this paper proposes the Enhanced Multi-Aspect Transformer (EMAT), a novel deep learning architecture specifically designed for stock market prediction. EMAT incorporates a Multi-Aspect Attention Mechanism that simultaneously captures temporal decay patterns, trend dynamics, and volatility regimes through specialized attention components. The model employs an encoder–decoder architecture with enhanced feed-forward networks utilizing SwiGLU activation, enabling superior modeling of complex non-linear relationships. Furthermore, we introduce a comprehensive multi-objective loss function that balances point-wise prediction accuracy with volatility consistency. Extensive experiments on multiple stock market datasets demonstrate that EMAT consistently outperforms a wide range of state-of-the-art baseline models, including various recurrent, hybrid, and Transformer architectures. Our ablation studies further validate the design, confirming that each component of the Multi-Aspect Attention Mechanism makes a critical and quantifiable contribution to the model’s predictive power. The proposed architecture’s ability to simultaneously model these distinct financial characteristics makes it a particularly effective and robust tool for financial forecasting, offering significant improvements in accuracy compared to existing approaches.

## 1. Introduction

Stock price forecasting represents one of the most critically important yet fundamentally challenging tasks in modern financial markets [1]. The accuracy of such predictions directly influences investment decision-making processes for individual investors through enhanced asset allocation strategies, while simultaneously serving as a cornerstone for corporate strategic planning and institutional risk management frameworks. The inherent complexity of this task stems from the highly non-linear and dynamic evolution of financial time series, which are shaped by a complex interplay of factors including historical price patterns, company-specific economic fundamentals, market sentiment dynamics, and broader macroeconomic conditions [2]. Financial markets exhibit intrinsic non-stationarity and extreme volatility, characteristics that render conventional forecasting approaches inadequate for capturing the full spectrum of market dynamics and pose fundamental challenges to predictive modeling.

To address these challenges, effective predictive models must demonstrate the capability to simultaneously capture short-term market fluctuations and long-term temporal dependencies inherent in financial time series data. The development of sophisticated architectures that can model complex temporal relationships while maintaining computational efficiency has thus become a central focus in advancing stock price prediction methodologies.

Early approaches to stock price modeling predominantly relied on classical statistical methods [3], including moving averages, ARMA, ARIMA, ARCH, and GARCH models. While these methods offer computational simplicity and interpretability, they fundamentally depend on idealized assumptions such as linearity and stationarity that are rarely satisfied in complex, real-world financial markets [4]. In practice, numerous dynamic uncertainties—including market noise, policy changes, and market manipulation—interact in complex ways, rendering traditional statistical models inadequate for extracting meaningful patterns from large-scale, high-dimensional market data.

Recognizing these limitations, researchers subsequently turned to machine learning algorithms [5], including K-Nearest Neighbors (KNN), decision trees, Support Vector Machines (SVM), random forests, and XGBoost. These approaches demonstrated improved performance by accommodating more flexible data distributions and have been successfully applied to various tasks including investment selection, feature extraction, and risk prediction [6]. However, traditional machine learning methods often rely heavily on handcrafted features and exhibit limited capacity to capture deep temporal patterns or complex interactions among multiple time series variables. Furthermore, they face significant challenges in scaling to and generalizing across complex, large-scale financial markets.

The advent of advanced computational resources has facilitated the development of deep learning approaches capable of extracting latent patterns from extensive historical financial data [7]. Models such as Convolutional Neural Networks (CNNs), Recurrent Neural Networks (RNNs), and Long Short-Term Memory networks (LSTMs) have gained prominence in financial forecasting applications. CNNs excel at capturing local spatial–temporal correlations and have been successfully applied to stock price direction prediction and multi-feature integration tasks [8]. RNNs inherently process sequential information but suffer from vanishing or exploding gradient problems when handling long sequences. LSTMs address these gradient issues through sophisticated gating mechanisms [9] and demonstrate superior capability in capturing long-term dependencies. Nevertheless, even LSTM-based methods can experience information decay over very long sequences due to their sequential processing nature, which limits their ability to fully capture evolving patterns in extended financial time series.

More recently, Transformer models—originally developed for natural language processing—have been increasingly adapted for time series forecasting applications [10,11,12]. Existing financial applications include enhancing locality awareness and integrating auxiliary data such as social media sentiment or textual news signals with price data. However, a critical limitation of many current Transformer-based approaches in finance is their primary focus on leveraging the architecture to analyze auxiliary inputs (e.g., social sentiment, news content) rather than fundamentally enhancing feature extraction from core numerical time series data [13]. This heavy reliance on external, often uncertain information sources can result in unstable performance and limited generalizability across different markets, particularly since the quality and relevance of such auxiliary data can vary significantly [14]. Consequently, many existing models emphasize data breadth through multi-modal inputs rather than achieving depth of understanding in the primary time series patterns. When core numerical feature extraction capabilities are inadequate, incorporating potentially noisy auxiliary data may not yield reliable performance improvements, suggesting that strengthening a model’s ability to extract comprehensive features directly from historical time series data provides a more robust foundation for financial forecasting.

A notable advancement toward addressing this limitation is the Series Decomposition Transformer with Period-correlation (SDTP) [15], which focuses on intrinsic patterns within price series by explicitly separating trend and seasonal components while leveraging inherent periodicity. SDTP demonstrated superior performance by concentrating on core numerical data rather than external information sources. However, even this promising direction reveals significant opportunities for further enhancement [16]. Current Transformer models, including SDTP, may not fully capture the complex interplay of temporal dependencies, dynamic market trends, and varying volatility regimes simultaneously. Additionally, existing approaches often operate on single series and may underutilize the multi-dimensional nature of financial time series characteristics. These limitations highlight the critical need for more sophisticated attention mechanisms that can explicitly model multiple aspects of financial data concurrently [17].

To address these fundamental limitations and provide a more robust foundation for financial time series forecasting, this paper proposes a novel deep learning architecture: the Enhanced Multi-Aspect Transformer (EMAT). EMAT is specifically designed for stock price prediction by leveraging intrinsic temporal patterns in historical price data through sophisticated attention mechanisms that avoid dependence on uncertain external information sources. The core innovations of EMAT include (1) a Multi-Aspect Attention Mechanism that simultaneously incorporates temporal decay patterns, trend dynamics, and volatility awareness; (2) a comprehensive multi-objective loss function that balances point-wise prediction accuracy with volatility consistency; and (3) a flexible encoder–decoder architecture that adapts to different forecasting scenarios. This approach represents a significant advancement in adapting Transformer architectures for financial time series prediction by focusing on the temporal, trend, and volatility characteristics inherent in price movements, thereby providing enhanced robustness and generalizability compared to approaches that rely on external information sources. The key contributions of this paper are as follows:

1. We propose EMAT, a novel Transformer-based architecture that integrates a Multi-Aspect Attention Mechanism with an enhanced loss function to effectively capture complex temporal dependencies in financial time series data.
2. We introduce a comprehensive attention framework that simultaneously considers temporal proximity effects, trend dynamics, and volatility patterns, enabling the model to adapt to different market regimes and capture multiple dimensions of price behavior.
3. We design a multi-objective loss function that optimizes both point-wise prediction accuracy and volatility consistency, providing more robust predictions that align with practical financial forecasting requirements.
4. We conduct extensive experiments on multiple stock market datasets, demonstrating that EMAT consistently outperforms state-of-the-art time series forecasting methods in terms of prediction accuracy and stability across diverse market conditions.

The remainder of this paper is organized as follows. Section 2 reviews related work in financial time series forecasting. Section 3 formulates the problem and provides necessary background. Section 4 presents the proposed EMAT methodology. Section 5 reports experimental results and analysis. Finally, Section 6 concludes the paper and discusses future directions.

## 2. Related Work

In recent decades, forecasting stock market trends has attracted growing attention due to the critical importance of financial markets and their pervasive impact on economic activity [18]. However, accurately predicting stock prices remains a formidable challenge due to the inherent complexity, high volatility, and non-stationarity characteristics of financial markets [19].

Stock price prediction is typically formulated as a time series forecasting problem: given a historical sequence $\left(x_{1},x_{2},\ldots ,x_{T}\right)$, the objective is to predict future values $\left(x_{T+1},x_{T+2},\ldots ,x_{T+k}\right)$. Methodologies for stock price forecasting can be broadly categorized into three main classes: classical statistical models, traditional machine learning models, and modern deep learning models, each representing an evolutionary advance in forecasting capabilities.

### 2.1. Statistical Models for Financial Time Series Forecasting

Classical statistical approaches form the foundation of time series analysis, employing mathematical models to generate empirical predictions from historical data. Representative methods include ARMA [20], ARIMA [21], ARCH [22], and GARCH models [23]. These models are valued for their interpretability and computational efficiency, making them widely adopted in financial applications.

However, statistical models rely on strong assumptions such as linearity and stationarity, which are frequently violated in real-world stock markets [24]. Financial markets involve numerous interacting variables, abrupt policy changes, and behavioral factors that create complex and noisy dynamics [25]. Consequently, these traditional approaches often struggle to capture the intricate correlations and evolving uncertainties present in large-scale financial systems.

### 2.2. Machine Learning Models for Financial Time Series Forecasting

Machine learning (ML) approaches have gained prominence in financial data analysis by moving beyond the rigid assumptions of classical statistical models. Notable examples include K-Nearest Neighbors (KNN) [26,27], decision trees [28,29], Support Vector Machines (SVM) [30,31], and random forests [32]. These models have been successfully applied to various financial tasks, including investment selection, rule induction from historical data, stock price movement forecasting, and risk prediction.

However, traditional ML models face significant limitations in financial applications. They typically require extensive feature engineering and may overlook important financial indicators due to their limited capacity for automatic feature extraction. While filter-based and voting-based feature selection methods have been proposed to identify relevant indicators for stock returns [33], these approaches still struggle to capture the full spectrum of market dynamics, particularly under complex and volatile market conditions [34].

### 2.3. Deep Learning Models for Financial Time Series Forecasting

With the rise of high-performance computing, deep learning has achieved remarkable success in many domains, offering novel solutions for stock prediction. CNNs have been effectively utilized for price direction prediction and integrating diverse stock-related information [35,36].

RNNs and their variants, particularly LSTM networks [9], are widely adopted for their ability to process sequential data and capture temporal dependencies. LSTMs address the vanishing gradient problems inherent in vanilla RNNs through gating mechanisms, making them suitable for longer-term forecasting [37]. However, LSTMs can still suffer from information loss over very long sequences due to their sequential processing nature, potentially limiting their ability to capture evolving patterns in extended time series [38]. Furthermore, alternative RNN paradigms such as Reservoir Computing, specifically Echo State Networks (ESN), have been proposed as a computationally efficient approach. ESNs have demonstrated strong performance in time series forecasting while drastically reducing training complexity and resources for training with similar performance [39].

Recently, Transformer-based models [40] have been increasingly applied to financial forecasting, leveraging their parallel processing capabilities and self-attention mechanisms. Early applications include variants that enhance locality with multi-scale Gaussian priors [41] and models integrating auxiliary data such as social media [42] and sentiment information [43]. However, many early Transformer-based approaches primarily focused on integrating external information rather than fundamentally enhancing feature extraction from core price series. This reliance on potentially noisy external data can lead to unstable performance and limited generalizability across diverse markets. A more promising direction emphasizes strengthening intrinsic feature extraction from historical price data. The Series Decomposition Transformer with Period-correlation (SDTP) model exemplifies this approach by explicitly separating trend and seasonal components while leveraging inherent periodicity [15]. By concentrating on core numerical patterns rather than uncertain external features, SDTP has demonstrated improved forecasting performance.

Despite these advances, significant challenges remain in tailoring Transformer architectures to financial time series. Current limitations include the need for more granular attention mechanisms that explicitly account for time decay, trend direction, and volatility patterns. Additionally, standard positional encodings may not adequately capture irregular time intervals common in financial markets. These observations highlight the need for specialized architectures capable of capturing the multifaceted nature of financial data, directly motivating our proposed EMAT model.

## 3. Problem Definition

Consider a multivariate financial time series $X={\left\{x_{t}\right\}}_{t=1}^{T}$, where $x_{t}\in R^{d}$ denotes the market state at time step *t* and *d* is the number of features. Given a fixed-length historical window $X_{t}=\left(x_{t-I+1},x_{t-I+2},\ldots ,x_{t}\right)\in R^{I\times d}$ with window size *I*, our goal is to learn a prediction function $f:R^{I\times d}\to R$ that estimates the next day’s closing price:

(1) $${\hat{x}}_{t+1}=f\left(X_{t}\right).$$

In adapting the Transformer architecture for this task, we define a “token” as the feature vector $x_{t}$ corresponding to a single time step. Thus, the input window $X_{t}$ is treated as a sequence of tokens, where each token encapsulates the complete market state at that point in time. This direct tokenization approach preserves the full granularity of the time series data. The prediction problem is formulated as a rolling-window regression, where the model is trained to minimize the error between the predicted value ${\hat{x}}_{t+1}$ and the actual value $x_{t+1}$ over the training period. After each forecast, the window shifts forward by one time step and the process repeats for the next prediction.

Formally, given a training set $D={\left\{\left(X_{t},x_{t+1}\right)\right\}}_{t=I}^{T-1}$, we aim to find the optimal parameters $\theta^{*}$ of the prediction function $f_{\theta }$ that minimizes the following objective:

(2) $$\theta^{*}=\underset{\theta }{arg min}\sum_{t=I}^{T-1}L\left(f_{\theta }\left(X_{t}\right),x_{t+1}\right)$$

where L is an appropriate loss function that measures the discrepancy between predictions and actual values. The main challenge lies in capturing both the short-term market dynamics and long-term temporal dependencies in $X_{t}$, while accounting for the non-stationary and noisy nature of financial time series.

### 3.1. Transformer Architecture Overview

The Transformer model, introduced by Vaswani et al. [40], has significantly advanced natural language processing and has been adapted for time series forecasting. The canonical Transformer architecture comprises an encoder and a decoder. The encoder transforms input sequences through successive layers: each layer applies input embeddings with positional encodings to capture sequential order, multi-head self-attention to capture dependencies across positions, position-wise feed-forward networks for feature transformation, and residual connections with layer normalization for stable training.

The decoder extends this structure with additional mechanisms: it employs masked self-attention to prevent the model from attending to future tokens during training, cross-attention to incorporate information from the encoder, and position-wise feed-forward networks, all coupled with residual connections and layer normalization. A key strength of Transformers is their ability to capture long-range dependencies by processing all input positions in parallel, thereby overcoming the sequential limitations of RNNs. This property is particularly valuable for financial time series, which often exhibit complex long-term temporal patterns. However, financial time series are characterized by multiple interacting dimensions that pose additional challenges for effective modeling.

### 3.2. Multi-Dimensional Characteristics of Financial Time Series

Financial time series exhibit complex multi-dimensional characteristics that are fundamental to accurate forecasting. Understanding and modeling these characteristics is crucial for developing effective prediction models. We identify three key dimensions that significantly influence price movements and must be simultaneously considered in any robust forecasting framework.

First, financial time series exhibit complex temporal dependencies where recent observations typically carry more predictive power than distant ones. This temporal decay effect can be modeled as

(3) $$w_{t}=\exp(-\lambda \cdot |t-t_{0}\left|\right)$$

where $w_{t}$ represents the influence weight of observation at time *t*, $t_{0}$ is the current time, and λ controls the decay rate. Additionally, financial data often exhibit periodic patterns at multiple time scales, including daily trading patterns, weekly cycles, and seasonal variations, which can be represented as

(4) $$x_{t}=\sum_{i=1}^{K}\alpha_{i}\sin\left(2\pi f_{i}t+\phi_{i}\right)+\epsilon_{t}$$

where $x_{t}$ represents the time series value at time *t*, *K* is the number of periodic components, $f_{i}$ denotes the frequency of the *i*-th component, $\alpha_{i}$ and $\phi_{i}$ are the amplitude and phase respectively, and $\epsilon_{t}$ represents the non-periodic residual component.

Second, financial markets exhibit distinct trend behaviors that influence future price movements. Trend patterns can be characterized by momentum effects, where

(5) $${\mathrm{Momentum}}_{t}=\frac{P_{t}-P_{t-n}}{P_{t-n}}$$

where $P_{t}$ represents the price at time *t* and *n* is the lookback period. These trend dynamics manifest as bullish or bearish market regimes, with different predictive patterns emerging during trending versus sideways market conditions.

Third, financial markets experience varying volatility regimes that significantly impact prediction accuracy. Volatility clustering, where high-volatility periods tend to be followed by high-volatility periods, can be mathematically represented by the following:

(6) $$\sigma_{t}^{2}=\omega +\alpha \epsilon_{t-1}^{2}+\beta \sigma_{t-1}^{2}$$

where $\sigma_{t}^{2}$ represents the conditional variance at time *t*, ω is the constant term, α and β are parameters controlling the impact of past squared residuals and past variances, respectively, and $\epsilon_{t-1}$ is the previous period’s residual. Different volatility regimes require adaptive modeling approaches, as prediction patterns that work in low-volatility environments may fail during high-volatility periods.

These three characteristics are often modeled independently in traditional approaches, failing to capture their complex interactions. The challenge lies in developing unified frameworks that can simultaneously account for temporal dependencies, trend dynamics, and volatility patterns while adapting to their time-varying nature. This limitation motivates our development of a Multi-Aspect Attention Mechanism that explicitly integrates these three dimensions into a coherent modeling framework.

### 3.3. Challenges in Financial Time Series Prediction

The multi-dimensional characteristics discussed above give rise to several fundamental challenges that distinguish financial time series prediction from conventional forecasting tasks. These challenges highlight the limitations of existing approaches and underscore the need for sophisticated modeling frameworks.

First, financial markets exhibit strong non-stationarity, where statistical properties such as mean, variance, and correlation structures evolve dynamically over time [44]. This characteristic renders traditional statistical models inadequate for capturing evolving market patterns. Second, financial data contains substantial noise arising from market microstructure effects, high-frequency trading activities, and information asymmetries, which can obscure underlying price signals and lead to prediction instability [45].

Third, price movements are influenced by factors operating across multiple time scales, from high-frequency fluctuations to long-term macroeconomic trends, necessitating models capable of capturing multi-resolution temporal patterns. Fourth, financial markets experience distinct regime changes corresponding to different economic conditions—such as bull versus bear markets and high- versus low-volatility periods—each requiring adaptive modeling approaches [46]. Fifth, financial markets exhibit time-varying multiscaling behavior [47,48,49,50,51], a well-established stylized fact of stock market dynamics [52,53,54]. This means the statistical properties of market fluctuations are dependent on the time scale of observation, and the nature of this scaling can shift dramatically between stable, efficient market periods and turbulent, crisis periods. This dynamic complexity, where the underlying “rules” of the market change across different time horizons, poses a significant challenge for predictive models that may assume a more stable data-generating process.

These challenges directly correspond to the three key dimensions identified earlier: capturing temporal proximity effects to address multi-scale dependencies, identifying and adapting to trend patterns to handle regime changes, and responding to volatility variations to manage non-stationarity and noise. Effective solutions must integrate these capabilities within a unified framework that ensures robustness across diverse market conditions while maintaining computational efficiency for practical applications.

## 4. Methodology

To effectively address the complex temporal patterns inherent in financial time series, we propose EMAT, a novel deep learning architecture. This section details its information processing mechanism, which transforms raw time series data into accurate predictions through a structured pipeline. The process begins by representing the input as a sequence of high-dimensional tokens enriched with positional encodings to preserve temporal order.

**Figure 1:**
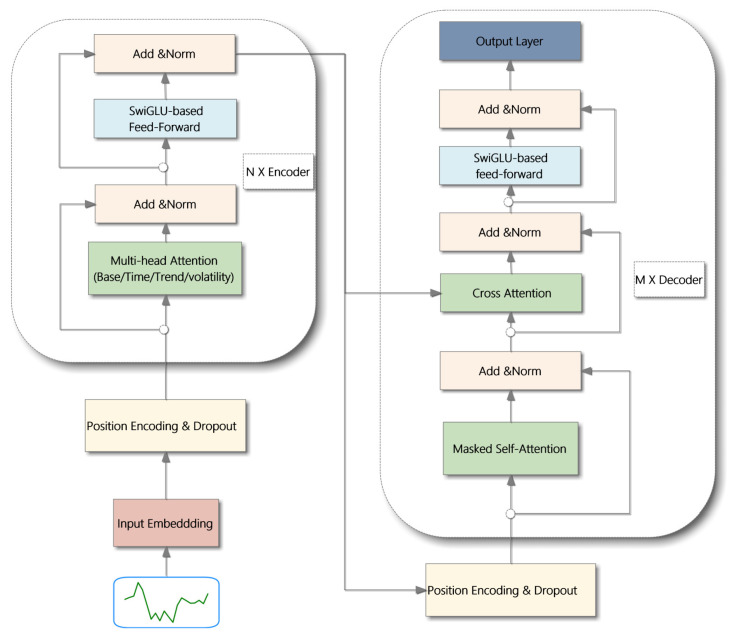
The architecture of the EMAT model.

At the core of the EMAT encoder is our Multi-Aspect Attention Mechanism, which simultaneously disentangles market signals from three distinct financial perspectives: temporal decay, trend dynamics, and volatility regimes. These parallel insights are then synergistically fused through a sequential gating process to create a holistic market representation. This enriched representation is subsequently utilized by the decoder, via cross-attention, to generate the final forecast. The entire predictive process is guided by a comprehensive multi-objective loss function that balances point-wise accuracy with volatility consistency. The following subsections provide the technical details of these components, and the overall architecture is illustrated in Figure 1.

### 4.1. Multi-Aspect Attention Mechanism

**Figure 2:**
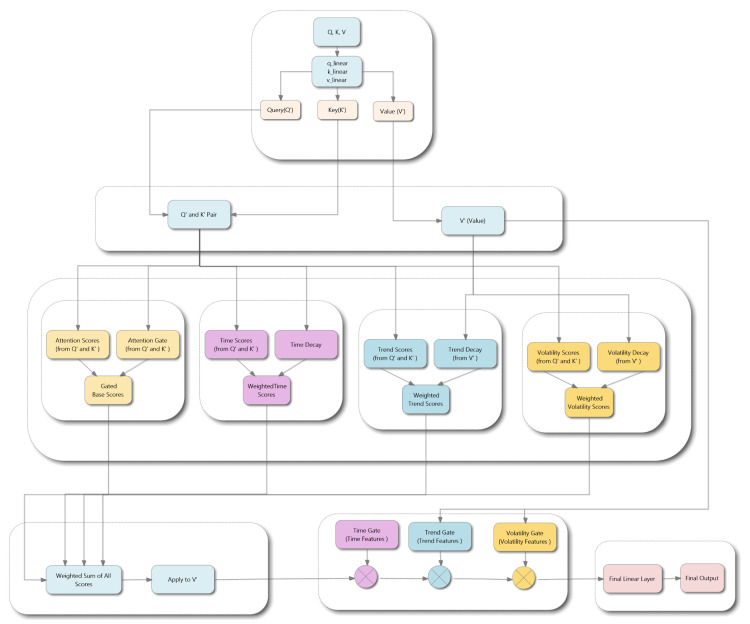
Architecture of the Multi-Aspect Attention Mechanism, showing base attention computation enhanced by temporal, trend, and volatility components, followed by sequential gating refinement.

Traditional multi-head attention mechanisms, while effective for many sequence modeling tasks, often fail to capture the complex temporal dynamics inherent in financial time series. To address this limitation, we propose a Multi-Aspect Attention Mechanism that enhances the standard attention framework by incorporating specialized components for temporal decay, trend analysis, and volatility awareness. The mechanism processes information through a base attention computation followed by three enhancement streams and sequential multiplicative gating for output refinement, as illustrated in Figure 2.

The mechanism begins by computing standard query (*Q*), key (*K*), and value (*V*) projections using linear transformations. These projections serve as inputs for the base attention computation and subsequent enhancement components.

#### 4.1.1. Base Attention with Content-Aware Gating

The foundation component computes scaled dot-product attention scores, which are then refined through a content-aware gating mechanism. The gated attention scores $A_{\mathrm{base}}$ are calculated as

(7) $$A_{\mathrm{base}}=\frac{QK^{T}}{\sqrt{d_{k}}}⊙{\mathrm{Gate}}_{\mathrm{content}}\left(\left[Q;K\right]\right)$$

where $d_{k}$ is the key dimension, ⊙ denotes element-wise multiplication, and ${\mathrm{Gate}}_{\mathrm{content}}$ is a neural network that processes concatenated query–key pairs to generate content-dependent attention modulation weights.

#### 4.1.2. Temporal Enhancement Component

This component captures temporal proximity effects by incorporating positional information and learnable decay patterns. The temporal enhancement is computed as

(8) $$A_{\mathrm{time}}=\frac{\left(QW_{q}^{\left(t\right)}\right){\left(KW_{k}^{\left(t\right)}\right)}^{T}}{\sqrt{d_{k}}}⊙{\mathrm{Decay}}_{\mathrm{time}}$$

where $W_{q}^{\left(t\right)}$ and $W_{k}^{\left(t\right)}$ are temporal projection matrices, and the decay matrix is defined as

(9) $${\mathrm{Decay}}_{\mathrm{time}}\left(i,j\right)=\exp(-|i-j|\cdot \sigma \left(\gamma_{t}\right))$$

with $\gamma_{t}$ being a learnable parameter and σ(·) the sigmoid function. Positional features are extracted through a dedicated neural network processing sequential position indices.

#### 4.1.3. Trend Enhancement Component

To capture market momentum patterns, this component analyzes price movement characteristics derived from value sequences. Price changes are computed as

(10) $$\Delta V_{t}=V_{t}-V_{t-1}$$

The computed price changes serve two critical functions in the trend-aware attention mechanism. First, they are processed through a dedicated feature extractor to generate trend-specific features:

(11) $${\mathrm{Features}}_{\mathrm{trend}}=f_{\mathrm{trend}}\left(\mathrm{mean}\left(\Delta V_{t}\right)\right)$$

where $f_{\mathrm{trend}}$ represents a neural network that extracts meaningful trend patterns from the average price changes.

Second, the price changes are utilized to compute a trend-based decay matrix that modulates attention based on the similarity of price movement patterns between different time steps:

(12) $${\mathrm{Decay}}_{\mathrm{trend}}\left(i,j\right)=\exp(-|\mathrm{mean}\left(\Delta V_{i}\right)-\mathrm{mean}\left(\Delta V_{j}\right)\left|\;\cdot \;\sigma \left(\gamma_{tr}\right)\right)$$

where $\gamma_{tr}$ is a learnable decay parameter.

The trend-aware attention component is then calculated as

(13) $$A_{\mathrm{trend}}=\frac{\left(QW_{q}^{\left(tr\right)}\right){\left(KW_{k}^{\left(tr\right)}\right)}^{T}}{\sqrt{d_{k}}}⊙{\mathrm{Decay}}_{\mathrm{trend}}$$

where $W_{q}^{\left(tr\right)}$ and $W_{k}^{\left(tr\right)}$ are trend-specific projection matrices. The extracted trend features ${\mathrm{Features}}_{\mathrm{trend}}$ are subsequently used in the sequential gating process to further refine the attention output.

#### 4.1.4. Volatility Enhancement Component

This component adapts attention patterns based on market volatility regimes. Volatility is computed using a sliding window approach:

(14) $${\mathrm{Vol}}_{i}=\mathrm{std}\left(V_{i-w+1:i}\right)$$

where *w* represents the window size (set to 5 in our implementation).

The computed volatility values serve two essential functions in the volatility-aware attention mechanism. First, they are processed through a dedicated feature extractor to generate volatility-specific features:

(15) $${\mathrm{Features}}_{\mathrm{vol}}=f_{\mathrm{vol}}\left({\mathrm{Vol}}_{i}\right)$$

where $f_{\mathrm{vol}}$ represents a neural network that extracts meaningful volatility regime patterns from the computed volatility values.

Second, the volatility information is utilized to compute a volatility-based decay matrix that modulates attention based on the similarity of volatility regimes between different time steps:

(16) $${\mathrm{Decay}}_{\mathrm{vol}}\left(i,j\right)=\exp(-|{\mathrm{Vol}}_{i}-{\mathrm{Vol}}_{j}\left|\;\cdot \;\sigma \left(\gamma_{v}\right)\right)$$

where $\gamma_{v}$ is a learnable decay parameter that controls the sensitivity to volatility differences.

The volatility-aware attention component is then calculated as

(17) $$A_{\mathrm{vol}}=\frac{\left(QW_{q}^{\left(v\right)}\right){\left(KW_{k}^{\left(v\right)}\right)}^{T}}{\sqrt{d_{k}}}⊙{\mathrm{Decay}}_{\mathrm{vol}}$$

where $W_{q}^{\left(v\right)}$ and $W_{k}^{\left(v\right)}$ are volatility-specific projection matrices. The extracted volatility features ${\mathrm{Features}}_{\mathrm{vol}}$ are subsequently employed in the sequential gating process to further modulate the attention output based on market volatility characteristics.

#### 4.1.5. Multi-Component Integration and Sequential Gating

The enhanced attention components are integrated with the base attention through learnable weight combinations:

(18) $$A_{\mathrm{combined}}=w_{\mathrm{base}}A_{\mathrm{base}}+w_{t}A_{\mathrm{time}}+w_{tr}A_{\mathrm{trend}}+w_{v}A_{\mathrm{vol}}$$

where the weights are derived from learnable parameters: $w_{t}=\sigma \left(\alpha_{t}\right)$, $w_{tr}=\sigma \left(\alpha_{tr}\right)$, $w_{v}=\sigma \left(\alpha_{v}\right)$, and $w_{\mathrm{base}}=1-w_{t}-w_{tr}-w_{v}$.

The combined attention scores are applied to the value matrix through softmax normalization:

(19) $${\mathrm{Output}}_{\mathrm{attn}}=\mathrm{softmax}\left(A_{\mathrm{combined}}\right)V$$

This intermediate output undergoes sequential refinement through multiplicative gating mechanisms:

(20) $$\begin{array}{cc} {\mathrm{Output}}_{1} & ={\mathrm{Output}}_{\mathrm{attn}}⊙{\mathrm{Gate}}_{\mathrm{time}}\left(\left[{\mathrm{Output}}_{\mathrm{attn}};{\mathrm{Features}}_{\mathrm{time}}\right]\right) \end{array}$$

(21) $$\begin{array}{cc} {\mathrm{Output}}_{2} & ={\mathrm{Output}}_{1}⊙{\mathrm{Gate}}_{\mathrm{trend}}\left(\left[{\mathrm{Output}}_{1};{\mathrm{Features}}_{\mathrm{trend}}\right]\right) \end{array}$$

(22) $$\begin{array}{cc} {\mathrm{Output}}_{\mathrm{final}} & ={\mathrm{Output}}_{2}⊙{\mathrm{Gate}}_{\mathrm{vol}}\left(\left[{\mathrm{Output}}_{2};{\mathrm{Features}}_{\mathrm{vol}}\right]\right) \end{array}$$

where each gate processes concatenated features to generate element-wise modulation weights. This multi-aspect approach enables the model to develop a comprehensive understanding of financial time series patterns, incorporating temporal proximity, trend dynamics, and volatility regimes into a unified attention framework. The proposed EMAT architecture, through its integration of specialized attention mechanisms and enhanced loss functions, provides a robust foundation for accurate financial time series prediction.

### 4.2. Enhanced Loss Function

Financial time series prediction requires a comprehensive loss function that captures multiple aspects of prediction accuracy. Traditional loss functions often focus solely on point-wise accuracy, which may not adequately reflect the complex nature of financial forecasting. To address this limitation, we propose a multi-objective loss function that simultaneously considers point-wise accuracy and volatility prediction. This enhanced loss function is designed to better align with the practical needs of financial forecasting and improve the model’s ability to capture various aspects of market behavior.

The total loss function is formulated as a weighted combination of three components:

(23) $$L_{\mathrm{total}}=\lambda_{1}L_{\mathrm{MSE}}+\lambda_{2}L_{\mathrm{MAE}}+\lambda_{3}L_{\mathrm{volatility}}$$

where $\lambda_{1},\lambda_{2},\lambda_{3}$ are hyperparameters that determine the relative importance of each component. These weights are set based on the characteristics of the target financial market.

The first component, mean squared error (MSE), measures the average squared difference between predicted and actual values:

(24) $$L_{\mathrm{MSE}}=\frac{1}{n}\sum_{i=1}^{n}{\left(y_{i}-{\hat{y}}_{i}\right)}^{2}$$

This component penalizes larger errors more heavily, making it particularly sensitive to outliers and extreme market movements. The MSE loss helps the model focus on reducing significant prediction errors, which is crucial for financial applications where large errors can have substantial implications.

The second component, mean absolute error (MAE), provides a more robust measure of prediction accuracy:

(25) $$L_{\mathrm{MAE}}=\frac{1}{n}\sum_{i=1}^{n}\left|y_{i}-{\hat{y}}_{i}\right|$$

Unlike MSE, MAE is less sensitive to outliers and provides a more balanced view of the model’s performance. This component helps ensure that the model maintains reasonable prediction accuracy across all data points, not just those with extreme values.

The third component, volatility loss, measures the accuracy of volatility predictions:

(26) $$L_{\mathrm{volatility}}=\frac{1}{m}\sum_{j=1}^{m}{\left(\sigma_{j}^{\mathrm{pred}}-\sigma_{j}^{\mathrm{true}}\right)}^{2}$$

where $\sigma_{j}^{\mathrm{pred}}$ and $\sigma_{j}^{\mathrm{true}}$ represent the predicted and actual volatility computed using sliding windows, respectively. The volatility is calculated as the standard deviation within a sliding window of size *w*:

(27) $$\sigma_{j}=\mathrm{std}\left(\mathrm{unfold}{\left(x,\mathrm{size}=w,\mathrm{step}\right)}_{j}\right)$$

where *w* denotes the window size, the unfold operation creates overlapping windows of size *w* with unit step, and the standard deviation is computed for each window. In our implementation, we set w=5 to capture short-term market fluctuations. This component enables the model to better capture market volatility patterns and adapt to different market regimes.

The implementation of this enhanced loss function involves several key steps. First, the input predictions and targets are flattened to ensure consistent dimensionality. The MSE and MAE components are computed using standard loss computation methods. For volatility calculation, we apply the unfold operation to create sliding windows of size *w*, then compute the standard deviation within each window for both predictions and targets. The volatility loss is then computed as the mean squared error between predicted and actual volatility sequences.

This enhanced loss function offers several advantages over traditional approaches. It balances point-wise accuracy with volatility prediction, considers both magnitude sensitivity through MSE and robustness through MAE, incorporates market volatility patterns through sliding window analysis, and provides a comprehensive evaluation metric that aligns with practical financial forecasting needs. By simultaneously optimizing multiple objectives, the model can better capture the complex nature of financial time series and provide more reliable predictions for practical applications.

### 4.3. Model Architecture

The EMAT model employs an encoder–decoder architecture tailored for financial time series forecasting. This structure enables the model to capture complex temporal dependencies by separating the roles of sequence representation and prediction generation. The model begins by processing the input sequence of tokens, where each token represents the feature vector of a single time step, as established in Section 3. An input embedding layer first transforms each token from its original feature dimension into a higher-dimensional vector representation. The encoder is composed of multiple layers, each containing a Multi-Aspect Attention Mechanism and an enhanced feed-forward network based on the SwiGLU activation. Specifically, each encoder layer adopts a pre-normalization residual formulation:

(28) $$\mathrm{EncoderLayer}\left(x\right)=x+\mathrm{Dropout}\left(\mathrm{MAAM}\left(\mathrm{LayerNorm}\left(x\right)\right)\right)+\mathrm{Dropout}\left(\mathrm{SwiGLU}\left(\mathrm{LayerNorm}\left(x^{\prime }\right)\right)\right)$$

where $x^{\prime }$ is the output of the first residual connection, MAAM denotes our Multi-Aspect Attention Mechanism, and SwiGLU is the enhanced feed-forward component. To preserve temporal information, positional encodings are incorporated as follows:

(29) $$x_{\mathrm{pos}}=x\sqrt{d_{\mathrm{model}}}+\mathrm{PE}$$

where PE denotes sinusoidal positional embeddings, and $d_{\mathrm{model}}$ is the embedding dimension.

The decoder utilizes both self-attention and cross-attention to integrate target-side and source-side information. Each decoder layer consists of three major components:

(30) $$\begin{array}{cc} y_{1} & =x+\mathrm{Dropout}(\mathrm{SelfAttention}(\mathrm{LayerNorm}(x))) \end{array}$$

(31) $$\begin{array}{cc} y_{2} & =y_{1}+\mathrm{Dropout}\left(\mathrm{CrossAttention}\left(\mathrm{LayerNorm}\left(y_{1}\right),\mathrm{enc}\text{\_}\mathrm{output}\right)\right) \end{array}$$

(32) $$\begin{array}{cc} y_{\mathrm{out}} & =y_{2}+\mathrm{Dropout}\left(\mathrm{SwiGLU}\left(\mathrm{LayerNorm}\left(y_{2}\right)\right)\right) \end{array}$$

where enc_output represents the encoder’s output. The decoder incorporates causal masking to maintain temporal integrity during training and incorporates both self-attention and cross-attention computations.

## 5. Experiments

To comprehensively evaluate the performance of the proposed EMAT model, we conduct extensive experiments following a systematic evaluation framework. Our experimental design encompasses several key components: dataset curation and preprocessing, evaluation metrics definition, baseline model comparison, detailed experimental setup, comprehensive performance analysis across diverse markets, ablation studies to assess individual component contributions, and parameter sensitivity analysis.

The evaluation framework follows a structured approach. First, we establish the experimental foundation through careful dataset selection and feature preprocessing, followed by the definition of appropriate evaluation metrics for financial time series prediction. We then benchmark EMAT against state-of-the-art baseline models using standardized experimental protocols. Performance analysis is conducted across two major market categories, namely Chinese and global market indices, ensuring comprehensive validation across diverse market conditions and geographic regions. Subsequently, ablation studies quantify the individual contributions of key architectural components, while parameter sensitivity analysis provides insights into model robustness and optimal configuration strategies.

This systematic approach ensures thorough validation of EMAT’s effectiveness while providing detailed insights into its behavior across different market conditions, time periods, and parameter configurations. The comprehensive evaluation demonstrates both the model’s superior predictive performance and its practical applicability for real-world financial forecasting scenarios.

### 5.1. Dataset and Preprocessing

For rigorous validation of the EMAT model, we curated a diverse collection of financial time series datasets spanning different geographic markets and market characteristics. The dataset selection strategy validates the model’s effectiveness across various market conditions, volatility regimes, and temporal patterns.

- Chinese Market Indices: We selected three major Chinese stock market indices representing different market segments: the Shanghai Stock Exchange Composite Index (SSE Composite, 000001.SS), the Shenzhen Stock Exchange Component Index (SZSE Component, 399001.SZ), and the China Securities Index 300 (CSI 300, 000300.SS). These indices provide comprehensive coverage of the Chinese equity market and represent different market capitalizations and sector compositions.
- Global Market Indices: To evaluate the model’s generalization capabilities across international markets, we include three major global indices: the Dow Jones Industrial Average (DJI), the S&P 500 Index, and the CAC 40 Index. These indices represent mature developed markets with distinct characteristics, allowing us to assess the model’s robustness across different economic environments and market structures.

**Figure 3:**
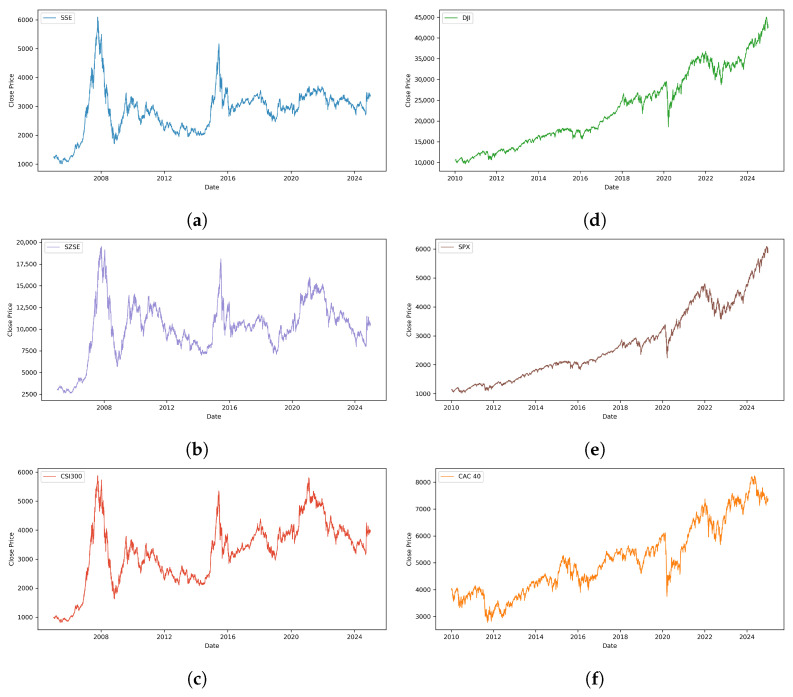
Historical closing price trends for all selected market indices. The (**left**) column displays Chinese market indices, while the (**right**) column displays global market indices. (**a**) SSE Composite; (**b**) SZSE Component; (**c**) CSI 300; (**d**) DJIA; (**e**) S&P 500; (**f**) CAC 40.

Table 1 provides an overview of the selected datasets, including their categorization, trading symbols, and key market characteristics. Figure 3 illustrates the historical price trajectories for all selected indices, demonstrating the diverse volatility patterns and trend behaviors across Chinese and global financial markets.

To ensure effective model training and consistent performance across different market conditions, we apply a rolling Min–Max normalization technique specifically designed for financial time series. Unlike traditional global normalization approaches, this method employs a sliding window mechanism that adapts to local market conditions:

(33) $$x_{i}^{*}=\frac{x_{i}-\min\left(x_{i-w:i-1}\right)}{\max\left(x_{i-w:i-1}\right)-\min\left(x_{i-w:i-1}\right)}$$

where $x_{i}$ represents the original price at time *i*, *w* denotes the rolling window size, and $x_{i}^{*}$ is the normalized value. This rolling normalization approach offers several advantages: (1) it adapts to changing market regimes and volatility levels; (2) it preserves local temporal relationships within the data; (3) it reduces the impact of long-term trends on short-term pattern recognition. The normalization window size is empirically set to ensure optimal balance between local adaptivity and statistical stability. During evaluation, predictions are inverse-transformed to their original price scale using the corresponding rolling statistics for accurate performance assessment.

### 5.2. Evaluation Metrics

To provide comprehensive and objective assessment of model performance, we employ four widely adopted evaluation metrics that capture different aspects of prediction accuracy in financial time series forecasting. These metrics collectively enable thorough comparison between EMAT and baseline methods while addressing the specific requirements of financial prediction tasks.

Given true values $y_{i}$ and predicted values ${\hat{y}}_{i}$ for *n* test samples, the evaluation metrics are defined as follows:

- Mean absolute error (MAE) measures the average magnitude of prediction errors:
  (34) $$\mathrm{MAE}=\frac{1}{n}\sum_{i=1}^{n}\left|y_{i}-{\hat{y}}_{i}\right|$$
- Root mean square error (RMSE) emphasizes larger errors through quadratic weighting:
  (35) $$\mathrm{RMSE}=\sqrt{\frac{1}{n}\sum_{i=1}^{n}{\left(y_{i}-{\hat{y}}_{i}\right)}^{2}}$$
- Mean absolute percentage error (MAPE) provides scale-independent relative error measurement:
  (36) $$\mathrm{MAPE}=\frac{100}{n}\sum_{i=1}^{n}\left|\frac{y_{i}-{\hat{y}}_{i}}{y_{i}}\right|$$
- Coefficient of determination ($R^{2}$) quantifies the proportion of variance explained by the model:
  (37) $$R^{2}=1-\frac{\sum_{i=1}^{n}{\left(y_{i}-{\hat{y}}_{i}\right)}^{2}}{\sum_{i=1}^{n}{\left(y_{i}-\bar{y}\right)}^{2}}$$
  where $\bar{y}=\frac{1}{n}\sum_{i=1}^{n}y_{i}$ represents the mean of true values.

These complementary metrics address distinct evaluation perspectives essential for financial forecasting. MAE provides robust assessment of average prediction accuracy with reduced sensitivity to extreme errors, making it particularly suitable for evaluating consistent model performance. RMSE emphasizes larger deviations through quadratic weighting, thereby capturing the model’s capacity to avoid significant mispredictions that could have substantial financial implications. MAPE enables scale-independent comparison across different price levels and market conditions through relative error measurement. Finally, $R^{2}$ quantifies the model’s explanatory power and goodness-of-fit to underlying data patterns.

For MAE, RMSE, and MAPE, lower values indicate superior predictive performance, with optimal values approaching zero. For $R^{2}$, values approaching 1.0 signify better model fit, with $R^{2}=1.0$ representing perfect prediction. This multi-metric evaluation framework ensures comprehensive assessment of model capabilities across different error characteristics and provides robust validation of the proposed EMAT architecture.

- LSTM [55]: LSTM networks are specialized RNNs designed to capture long-range dependencies in sequential data. They employ gating mechanisms (input, output, and forget gates) to regulate information flow, thereby mitigating vanishing gradient problems. While effective for time series modeling, their sequential processing nature can be computationally intensive and may suffer from information decay over extremely long sequences.
- BiLSTM [56]: Bidirectional LSTMs enhance standard LSTMs by processing input sequences in both forward and backward directions, enabling the capture of contextual information from past and future states. This bidirectional approach improves prediction accuracy in financial forecasting, though often at increased computational cost.
- GRU [57]: Gated Recurrent Units simplify LSTM architecture by combining forget and input gates into a single update gate and merging cell and hidden states. This design yields computationally efficient models with fewer parameters, making GRUs attractive alternatives for sequence modeling tasks.
- CNN-LSTM [58]: This hybrid architecture combines CNNs with LSTMs, where CNN layers extract local patterns through one-dimensional convolutions, and LSTM layers model temporal dynamics of extracted features. This combination leverages both local feature extraction capabilities and sequential modeling strengths.
- CNN-BiLSTM [59]: Building upon CNN-LSTM, this architecture replaces unidirectional LSTMs with BiLSTMs, allowing temporal modeling components to leverage bidirectional context from CNN-extracted features for enhanced pattern understanding.
- CNN-BiLSTM-AM [59]: This model incorporates attention mechanisms into the CNN-BiLSTM framework. The attention layer dynamically assigns weights to hidden states across different time steps, enabling focus on influential historical patterns and improving prediction accuracy.
- Transformer [60]: This architecture relies entirely on self-attention mechanisms, processing input points in parallel to model global dependencies regardless of sequential distance. While offering computational advantages and superior long-range interaction modeling, standard Transformers are domain-agnostic and may not be optimized for financial time series characteristics such as volatility and trend dynamics.

### 5.3. Experimental Setup

To comprehensively evaluate the effectiveness of EMAT, we conduct comparative experiments following a systematic protocol. All experiments were conducted on a standardized computing platform using a PyTorch 2.5 deep learning framework. To ensure reproducibility, we fixed random seeds across all experiments and maintained consistent computational environments.

For each dataset, we follow a systematic training protocol with models trained on designated training sets, the time ranges of which are detailed in Table 2. The optimal hyperparameters for EMAT were determined through multiple training sessions and empirical evaluation, as summarized in Table 3. All baseline models are configured with their respective optimal hyperparameters to ensure fair comparison.

Model performance is assessed using the four evaluation metrics described in the Evaluation Metrics section on designated test sets. To ensure statistical reliability, we conduct multiple training runs with different random initializations and report average performance. The reported results represent performance on completely unseen test data, maintaining strict separation between training and testing phases to prevent data leakage. All models are trained for a fixed number of epochs to ensure optimal performance.

### 5.4. Performance Comparison and Analysis

To validate the effectiveness and robustness of EMAT model, we conducted a detailed performance comparison against baseline models across all datasets. The results are presented and analyzed by category in the following subsections.

#### 5.4.1. Results on Chinese Market Indices

The experimental results for the three major Chinese market indices are presented in Table 4 and Table 5. EMAT demonstrates consistent superior performance across all evaluation metrics compared to competitive baselines, including recurrent architectures (LSTM, BiLSTM, GRU), hybrid models (CNN-LSTM, CNN-BiLSTM, CNN-BiLSTM-AM), and the Transformer.

On the SSE Composite Index, EMAT achieves a mean absolute error of 24.2440 and a root mean square error of 34.9370. These results represent improvements over the Transformer model, which attains 24.6510 in MAE and 35.5720 in RMSE, corresponding to relative error reductions of approximately 1.65% and 1.78%, respectively.

For the SZSE Component Index, EMAT yields the lowest errors among all methods, with an MAE of 111.3750 and an RMSE of 157.0250. On the CSI 300 Index, the model achieves an MAE of 33.7990 and an RMSE of 48.3970, demonstrating its ability to generalize across multiple markets. Furthermore, EMAT records a mean absolute percentage error of 0.8722% and an $R^{2}$ value of 0.9804, indicating excellent predictive accuracy.

These results highlight the model’s effectiveness in capturing temporal dependencies in financial time series. The high $R^{2}$ values across all indices—such as the 0.9591 observed for the SSE Composite—underscore the model’s strong explanatory power and its capability to reconstruct actual market movements with high fidelity.

**Figure 4:**
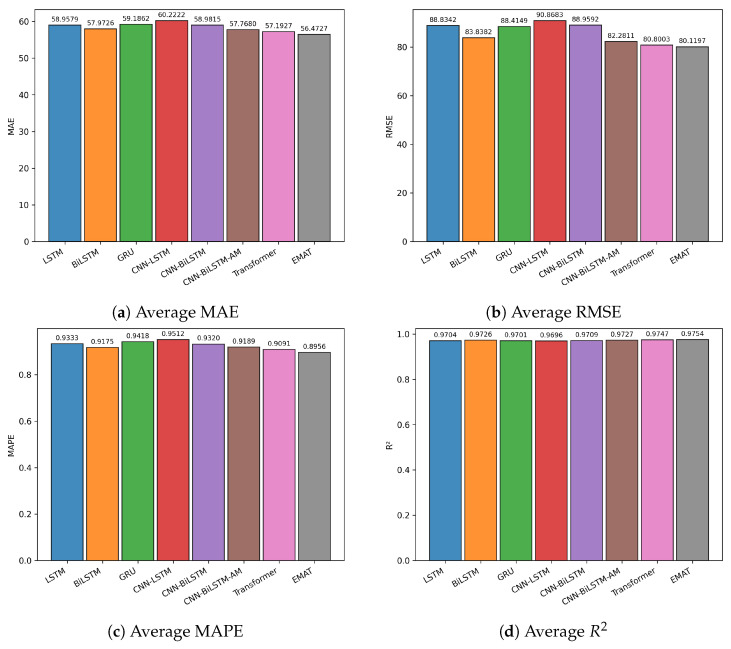
Bar chart visualization of average evaluation metrics across the three Chinese market indices. Each subplot corresponds to a different metric: (**a**) MAE, (**b**) RMSE, (**c**) MAPE, and (**d**) $R^{2}$.

**Figure 5:**
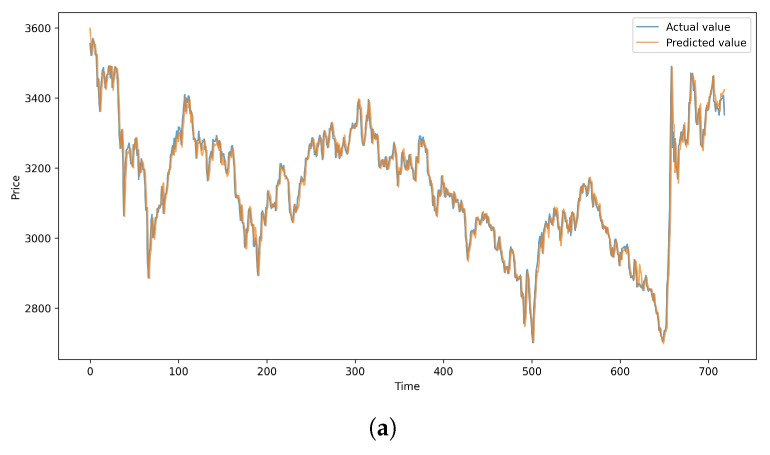

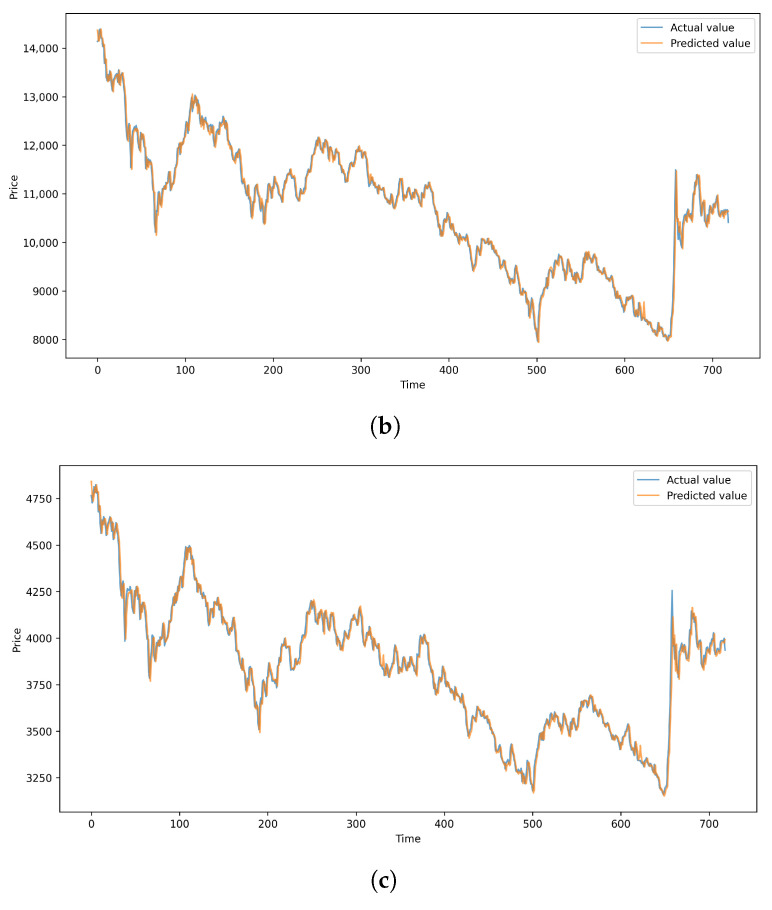
Comparison between the predicted (red) and actual (blue) closing prices on the test sets for the three major Chinese market indices. The predictions from our EMAT model closely track the true price movements. (**a**) SSE Composite Index; (**b**) SZSE Component Index; (**c**) CSI 300 Index.

Figure 4 provides a comparative visualization of performance across the three indices, while Figure 5 shows the close alignment between predicted and actual closing prices. Together, these results demonstrate the robustness and accuracy of EMAT in real-world stock market forecasting tasks.

#### 5.4.2. Results on Global Markets Indices

To evaluate the generalizability of the proposed EMAT model beyond the Chinese stock market, we conduct experiments on three representative global stock indices: DJIA, the S&P 500, and the CAC 40. The quantitative results, reported in Table 6 and Table 7, confirm EMAT’s superior performance across diverse market conditions and geographic regions.

EMAT demonstrates consistent superior performance across all global market indices compared to baseline methods. On the DJIA index, EMAT achieves a mean absolute error of 240.3570 and a root mean square error of 322.8700, outperforming the best baseline, Transformer, which yields 246.1640 in MAE and 329.0910 in RMSE. For the S&P 500, EMAT attains the lowest errors, with an MAE of 36.8150 and an RMSE of 48.7510. On the CAC 40 index, EMAT also delivers superior results, achieving an MAE of 55.3640 and an RMSE of 74.4850. These results indicate that EMAT offers improved predictive accuracy on a global scale.

In terms of relative percentage errors, EMAT achieves the lowest MAPE values across all three indices. For example, the MAPE on the S&P 500 is 0.8303%. In addition, the model produces the highest $R^{2}$ scores among all evaluated methods. Specifically, the $R^{2}$ value reaches 0.9923 for DJIA, 0.9946 for the S&P 500, and 0.9840 for CAC 40. These results indicate strong explanatory power and minimal variance in prediction errors.

**Figure 6:**
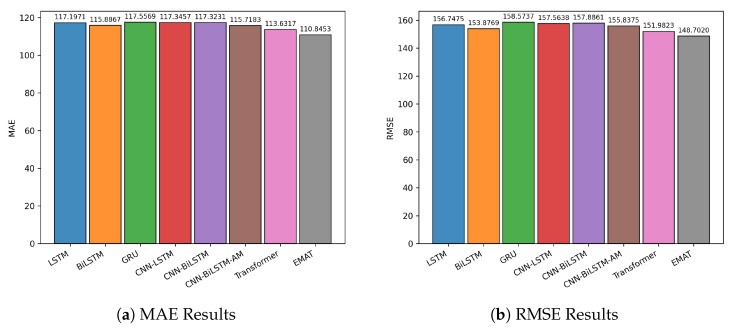

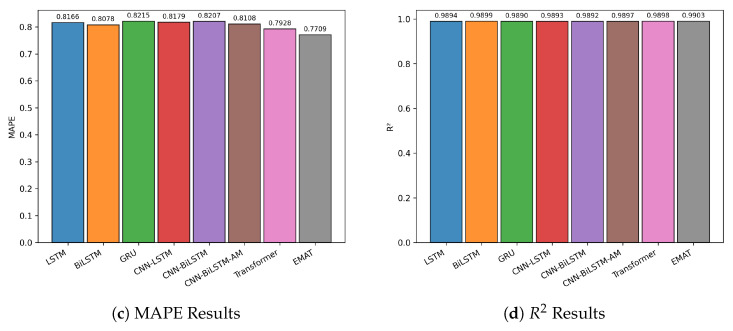
Bar chart visualization of evaluation metrics for the proposed EMAT model on the three major global market indices. Each subplot corresponds to a different metric: (**a**) MAE, (**b**) RMSE, (**c**) MAPE, and (**d**) $R^{2}$.

**Figure 7:**
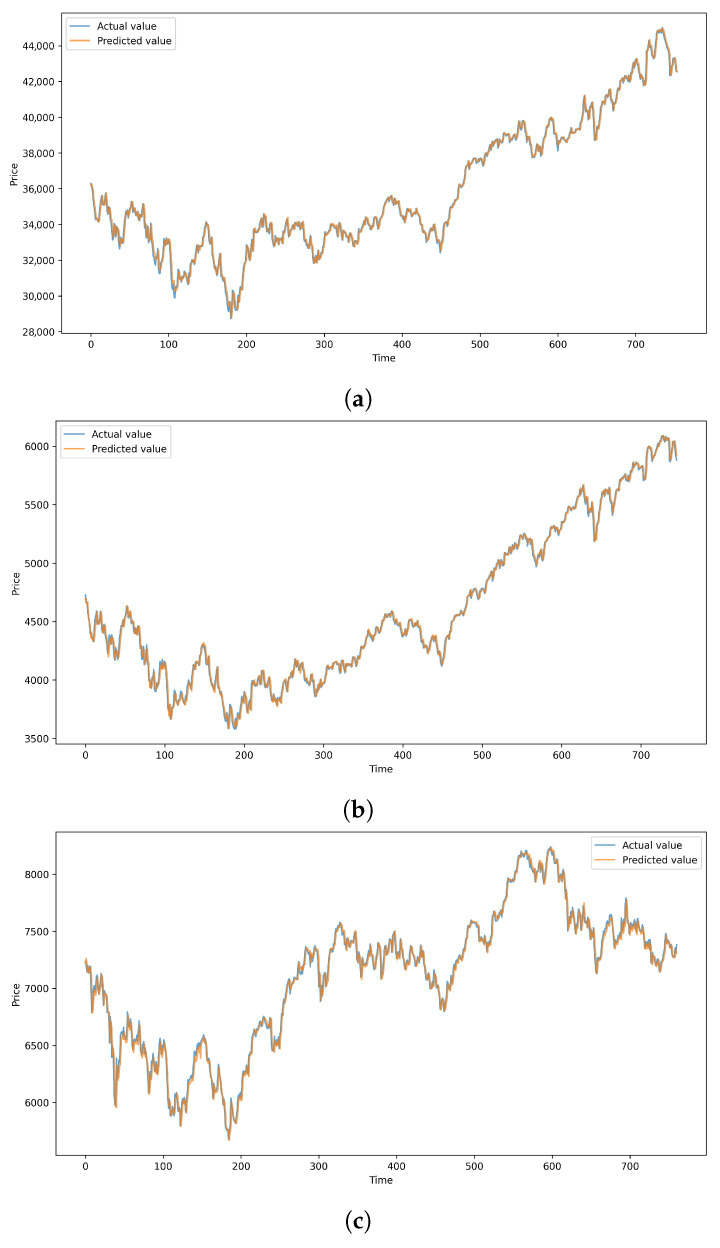
Comparison between the predicted (red) and actual (blue) closing prices on the test sets for the three major global market indices. (**a**) DJIA; (**b**) S&P 500; (**c**) CAC 40.

Figure 6 provides a comparative visualization of the average performance across all evaluation metrics, and Figure 7 illustrates the close alignment between EMAT’s predicted values and the actual index prices. These results confirm EMAT’s robustness across diverse market structures and trading environments. The consistent performance improvements across both Chinese and global markets validate the generalizability of the proposed Multi-Aspect Attention Mechanism for financial time series forecasting.

### 5.5. Ablation Study

To validate the effectiveness and generalizability of the key components within our proposed model, we conducted a series of ablation experiments. The study was performed on two representative indices: the SSE Composite Index from the Chinese market and the S&P 500 from the global market. We systematically tested the impact of our core architectural choices by comparing the full EMAT model with several variants where individual components were removed. The results of this analysis are reported in Table 8.

The experiments clearly demonstrate the contribution of each specialized feature in our enhanced attention mechanism. The variant EMAT w/o Time, which lacks the time-aware component, shows a noticeable degradation in performance across all metrics for both indices. A similar decline is observed for the EMAT w/o Trend and EMAT w/o Volatility variants. For the SSE Composite Index, removing any single component increases the MAE, MAPE, and RMSE values while decreasing the $R^{2}$ score. This confirms that each architectural component provides a unique and valuable contribution to the prediction task.

The consistency of these findings across both the SSE Composite and S&P 500 datasets is particularly significant. It strongly indicates that each component of our model contributes effectively and synergistically to the final predictive power. Furthermore, the benefits of our Multi-Aspect Attention Mechanism are generalizable across different market structures. The full EMAT model consistently outperforms all ablated versions, confirming that the integration of time, trend, and volatility awareness is essential to achieving state-of-the-art performance.

### 5.6. Parameter Sensitivity Study

To assess the sensitivity of the EMAT model to its hyperparameters, we conducted an analysis on a key parameter: the input sequence length. The length of the historical data fed into the model directly impacts the final forecast results. The sequence must be long enough to capture relevant patterns but short enough to avoid introducing irrelevant historical noise. To analyze the effect of different lag lengths, we performed experiments on the SSE Composite and the S&P 500 indices, with the results presented in Table 9.

As shown in the table, a lag length of 5 days, which was used in our main experiments, yields the best performance for both markets. When the lag length is increased to 7 days and subsequently to 10 days, there is a consistent degradation in performance across all evaluation metrics for both indices. For example, the MAE for the S&P 500 increases from 36.8150 at a lag of 5 days to 37.7600 at a lag of 10 days. This trend suggests that for these specific markets, a shorter lookback period is more effective at capturing predictive signals. The optimal selection of the lag length is crucial for improving model performance, and these results validate our choice of a 5-day input sequence for the primary model configuration.

## 6. Conclusions

In this paper, we proposed EMAT, a novel deep learning architecture designed to address the significant challenges of stock market prediction. Our work confronts the limitations of standard models that are often domain-agnostic and fail to capture the unique, multifaceted characteristics of financial time series. The EMAT model introduces a specialized Multi-Aspect Attention Mechanism that simultaneously integrates temporal, trend, and volatility information, complemented by a multi-objective loss function to enhance predictive stability.

To empirically validate the effectiveness of the proposed EMAT architecture and its constituent mechanisms, we conducted extensive experiments on multiple stock market datasets. The EMAT model consistently outperformed a wide range of state-of-the-art baseline methods, including various recurrent, hybrid, and Transformer architectures, demonstrating significant improvements in key evaluation metrics. Furthermore, our ablation studies confirmed the critical contribution of each component within the enhanced attention framework. The removal of any single aspect, be it temporal, trend, or volatility awareness, resulted in a quantifiable degradation of performance, proving the synergistic effectiveness of our design.

Collectively, these findings underscore the practical value and theoretical significance of our approach. Building on these results, this research demonstrates that by tailoring the Transformer architecture with domain-specific mechanisms, it is possible to achieve a new level of performance in stock price forecasting. The EMAT model provides a more robust and accurate tool for financial analysis. Future work could explore the application of this architecture to other financial instruments, such as commodities or cryptocurrencies, and investigate the integration of additional market factors within this multi-aspect framework. A particularly promising direction would be to incorporate the multiscaling characteristics of stock market time series into our Multi-Aspect Attention Mechanism to potentially enhance prediction accuracy.

## Figures and Tables

**Table 1:** Composition of experimental datasets.

Category	Name	Symbol	Key Characteristics
Chinese Market	SSE Composite Index	000001.SH	Benchmark index for Shanghai Stock Exchange
	SZSE Component Index	399001.SZ	Major index for Shenzhen-listed stocks
	CSI 300 Index	000300.SH	Tracks top 300 large-cap A-shares from SSE and SZSE
Global Markets	DJIA (Dow Jones)	DJI	30 major U.S. industrial companies
	S&P 500 Index	^GSPC	Broad-based U.S. large-cap equity index
	CAC 40 Index	^FCHI	Tracks 40 largest companies listed on Euronext Paris

**Table 2:** Training and testing time ranges for all datasets.

Index	Training Set Range	Test Set Range
SSE Composite	1 January 2005–31 December 2021	1 January 2022–31 December 2024
SZSE Component	1 January 2005–31 December 2021	1 January 2022–31 December 2024
CSI 300	1 January 2005–31 December 2021	1 January 2022–31 December 2024
DJIA	1 January 2010–31 December 2021	1 January 2022–31 December 2024
S&P 500	1 January 2010–31 December 2021	1 January 2022–31 December 2024
CAC 40	1 January 2010–31 December 2021	1 January 2022–31 December 2024

**Table 3:** EMAT model configuration and hyperparameters.

Category	Parameter	Value
Training Setup	Epochs	100
	Batch Size	64
	Learning Rate	0.001
	Optimizer	AdamW
Model Architecture	Encoder Layers	4
	Decoder Layers	4
	Attention Heads	8
	Model Dimension	256
	Dropout Rate	0.1
Loss Function	MSE Weight ($\lambda_{1}$)	0.5
	MAE Weight ($\lambda_{2}$)	0.4
	Volatility Weight ($\lambda_{3}$)	0.1

**Table 4:** Comparison of MAE and MAPE on Chinese market indices.

Model	MAE			MAPE (%)		
	**SSE** **Composite**	**SZSE** **Component**	**CSI** **300**	**SSE** **Composite**	**SZSE** **Component**	**CSI** **300**
LSTM	25.1222	116.8615	34.8901	0.7986	1.1004	0.9009
BiLSTM	24.4306	114.9543	34.5329	0.7774	1.0821	0.8929
GRU	25.6517	116.8241	35.0829	0.8159	1.1019	0.9076
CNN-LSTM	25.4385	119.6550	35.5731	0.8086	1.1283	0.9167
CNN-BiLSTM	25.0634	117.2090	34.6722	0.7968	1.1048	0.8945
CNN-BiLSTM-AM	25.0359	113.7938	34.4744	0.7958	1.0703	0.8905
Transformer	24.6510	112.6350	34.2920	0.7844	1.0581	0.8847
EMAT (Ours)	24.2440	111.3750	33.7990	0.7705	1.0441	0.8722

**Table 5:** Comparison of RMSE and $R^{2}$ on Chinese market indices.

Model	RMSE			$R^{2}$		
	**SSE** **Composite**	**SZSE** **Component**	**CSI** **300**	**SSE** **Composite**	**SZSE** **Component**	**CSI** **300**
LSTM	36.9929	177.6863	51.8235	0.9526	0.9821	0.9764
BiLSTM	36.1131	165.7673	49.6341	0.9548	0.9845	0.9784
GRU	37.4966	176.0028	51.7454	0.9513	0.9825	0.9765
CNN-LSTM	37.2077	182.5438	52.8534	0.9521	0.9811	0.9755
CNN-BiLSTM	36.5473	179.5091	50.8212	0.9537	0.9817	0.9774
CNN-BiLSTM-AM	36.4000	160.9999	49.4435	0.9541	0.9854	0.9785
Transformer	35.5720	158.0600	48.7690	0.9576	0.9865	0.9801
EMAT (Ours)	34.9370	157.0250	48.3970	0.9591	0.9866	0.9804

**Table 6:** Comparison of MAE and MAPE on global market indices.

Model	MAE			MAPE (%)		
	**DJIA**	**S&P** **500**	**CAC 40**	**DJIA**	**S&P 500**	**CAC 40**
LSTM	254.2116	39.4562	57.9234	0.7266	0.8904	0.8327
BiLSTM	251.4519	39.6921	56.5162	0.7162	0.8946	0.8127
GRU	254.6680	39.5423	58.4603	0.7284	0.8934	0.8426
CNN-LSTM	254.9555	39.7881	57.2935	0.7293	0.8996	0.8248
CNN-BiLSTM	254.4343	40.1760	57.3591	0.7291	0.9073	0.8258
CNN-BiLSTM-AM	250.2073	39.8113	57.1363	0.7186	0.8941	0.8197
Transformer	246.1640	37.9040	56.8270	0.7045	0.8562	0.8178
EMAT (Ours)	240.3570	36.8150	55.3640	0.6862	0.8303	0.7961

**Table 7:** Comparison of RMSE and $R^{2}$ on global market indices.

Model	RMSE			$R^{2}$		
	**DJIA**	**S&P 500**	**CAC 40**	**DJIA**	**S&P 500**	**CAC 40**
LSTM	341.1555	51.9819	77.1051	0.9915	0.9939	0.9829
BiLSTM	334.4265	52.1767	75.0275	0.9919	0.9939	0.9838
GRU	343.9346	52.5293	79.2573	0.9914	0.9938	0.9819
CNN-LSTM	342.3611	52.7512	77.5791	0.9915	0.9938	0.9827
CNN-BiLSTM	342.5598	52.9557	78.1429	0.9915	0.9937	0.9824
CNN-BiLSTM-AM	340.0012	51.9964	75.5148	0.9916	0.9939	0.9836
Transformer	329.0910	50.2940	76.5620	0.9921	0.9943	0.9830
EMAT (Ours)	322.8700	48.7510	74.4850	0.9923	0.9946	0.9840

**Table 8:** Ablation study results on the SSE Composite and S&P 500 sets.

Model Variation	SSE Composite				S&P 500			
	**MAE**	**MAPE (%)**	**RMSE**	$R^{2}$	**MAE**	**MAPE (%)**	**RMSE**	$R^{2}$
EMAT w/o Time	24.4820	0.7776	35.1430	0.9586	37.2737	0.8405	49.2145	0.9945
EMAT w/o Trend	24.3460	0.7734	35.1140	0.9587	37.1060	0.8369	49.1220	0.9945
EMAT w/o Volatility	24.4300	0.7764	35.0540	0.9588	37.1630	0.8373	49.2560	0.9945
EMAT (Full Model)	24.2440	0.7705	34.9370	0.9591	36.8150	0.8303	48.7510	0.9946

**Table 9:** Parameter sensitivity of EMAT model on representative indices with different input sequence lengths.

Lag Length	SSE Composite				S&P 500			
	**MAE**	**RMSE**	**MAPE (%)**	$R^{2}$	**MAE**	**RMSE**	**MAPE (%)**	$R^{2}$
Lag_10	24.5050	0.7793	35.1760	0.9572	37.7600	0.8509	50.1210	0.9944
Lag_7	24.4100	0.7763	34.8880	0.9586	37.6100	0.8478	49.7950	0.9944
Lag_5 (original)	24.2440	0.7705	34.9370	0.9591	36.8150	0.8303	48.7510	0.9946

## Data Availability

The data presented in this study are available on request from the corresponding author due to ethical and privacy restrictions. For detailed information, please contact the corresponding author.
